# Effects of statin therapy in hospitalized adult COVID-19 patients: a systematic review and meta-analysis of randomized controlled trials

**DOI:** 10.31744/einstein_journal/2023RW0351

**Published:** 2023-06-06

**Authors:** Débora Pinheiro Xavier, Gabriel Cavalcante Lima, Lorena Gisele Ferreira Gomes, Juliana Ferri-Guerra, Rafael Enrique Hernandez Oquet

**Affiliations:** 1 Department of Medicine Universidade Federal do Pará Belém PA Brazil Department of Medicine, Universidade Federal do Pará, Belém, PA, Brazil.; 2 Department of Internal Medicine Universidade Federal do Ceará Fortaleza CE Brazil Department of Internal Medicine, Universidade Federal do Ceará, Fortaleza, CE, Brazil.; 3 Department of Internal Medicine Mount Sinai Medical Center Miami Beach FL USA Department of Internal Medicine, Mount Sinai Medical Center, Miami Beach, FL, USA.; 4 Division of Hospital Medicine University of Miami Miller School of Medicine FL USA Division of Hospital Medicine, University of Miami Miller School of Medicine, FL, USA.

**Keywords:** COVID-19, Coronavirus infections, SARS CoV-2, Hydroxymethylglutaryl-CoA reductase inhibitors, Atorvastatin

## Abstract

**Introduction:**

COVID-19 is associated with endothelial activation and systemic inflammation; consequently, statins can be used in its treatment as they have anti-inflammatory, antithrombotic, and profibrinolytic properties and may interfere with COVID-19 viral entry into cells through disruption of cell membrane lipid rafts.

**Objective:**

We performed a meta-analysis of randomized clinical trials that compared statin therapy to placebo or to standard care in adult patients hospitalized for COVID-19.

**Methods:**

We searched the MEDLINE, EMBASE, and Cochrane Library databases for all-cause mortality, hospitalization duration, and admission to the intensive care unit.

**Results:**

Of the 228 studies reviewed, four studies were included, with a total of 1,231 patients, of whom 610 (49.5%) were treated with statins. There was no significant difference in all-cause mortality (odds ratio [OR] 0.96; 95% confidence interval [95%CI]: 0.61-1.51; p=0.86; I^2^=13%), duration of hospitalization (mean difference [MD] 0.21; 95%CI: -1.74-2.16; p=0.83; I^2^=92%), intensive care unit admission (OR= 3.31; 95%CI: 0.13-87.1; p=0.47; I2=84%), need for mechanical ventilation (OR= 1.03; 95%CI: 0.36-2.94; p=0.95; I^2^=0%), or increase in liver enzyme levels (OR= 0.58; 95%CI: 0.27-1.25; p=0.16; I^2^=0%) between patients treated with or without statin therapy.

**Conclusion:**

Our findings suggest that in adult patients hospitalized with COVID-19, statin therapy results in no difference in clinical outcomes when compared to outcomes by placebo or standard of care. **Prospero database registration:** (www.crd.york.ac.uk/prospero) under the number CRD42022338283.

## INTRODUCTION

Severe acute respiratory syndrome coronavirus 2 or coronavirus disease 2019 (SARS-CoV-2 or COVID-19) is an enveloped positive-sense RNA virus that invades host cells mainly through the angiotensin-converting enzyme 2 (ACE2) receptor and triggers an intense inflammatory response.^(1,2)^ Cytokine storm is thought to be a major contributor to disease severity and mortality.^(3-5)^

As of June 26, 2022, there have been more than 548 million confirmed cases of COVID-19 and more than 6 million deaths globally according to the World Health Organization (WHO).^(6)^ Despite the advances in vaccination, there is an urgent need to improve therapeutic options, especially in cases requiring hospitalization.

Recently, statins have emerged as a potential new therapy for patients with COVID-19 owing to their pleiotropic effects on inflammation and oxidative stress.^(7,8)^ They modulate the immune response at different levels, including immune cell adhesion and migration, antigen presentation, and cytokine production, while also exhibiting antithrombotic and profibrinolytic activities.^(9)^ Statins may also inhibit SARS-CoV-2 entry into the host cells by directly binding to the main viral protease enzyme.^(10)^ Given their widespread availability and optimal tolerability profile^(11)^ statins could be an effective and low-cost treatment alternative, particularly in developing countries. However, statins upregulate the expression of ACE2 in animal models, raising concerns about whether patients on statin therapy are at a greater risk for SARS-CoV-2 infection or worse outcomes.^(12-15)^

A previous meta-analysis suggested a reduced all-cause mortality in patients hospitalized with COVID-19 who were treated with statins.^(16)^ However, the pooled data were limited by the inclusion of observational studies, which are invariably prone to confounding factors. Considering the publication of multiple randomized controlled trials (RCTs) addressing this issue,^(17-20)^ we aimed to perform a systematic review and meta-analysis to assess the effects of statin therapy on the clinical outcomes of patients with COVID-19, restricted to randomized data.

## METHODS

We predefined our search strategy, study eligibility criteria, and statistical approaches for this systematic review and meta-analysis according to the Cochrane Handbook for Systematic Reviews of Interventions version 6.3, 2022^(21)^ and the Preferred Reporting Items for Systematic Reviews and Meta-Analysis (PRISMA) 2020 statement guidelines.^(22)^ This systematic review was prospectively registered with the International Prospective Register of Systematic Reviews (PROSPERO) on June 14, 2022.

### Eligibility criteria

We included RCTs comparing statin drugs (*e.g*., atorvastatin, fluvastatin, lovastatin, pitavastatin, pravastatin, rosuvastatin, and simvastatin) with placebo or standard of care in hospitalized adult patients (age ≥16 years) with COVID-19, and reported the outcomes of interest. We excluded studies with overlapping patient populations, studies with no control groups, head-to-head comparisons of statin drugs, and studies with samples randomized to combination therapy as an intervention. The main outcomes of interest were all-cause mortality, hospitalization duration, and admission to the intensive care unit (ICU). Additional outcomes of interest were the need for mechanical ventilation and an increase in liver enzyme levels (more than three times the upper limit of normal).

### Search strategy and data extraction

We conducted a systematic literature search of the Medical Literature Analysis and Retrieval System Online (MEDLINE), Excerpta Medica Database (EMBASE), and Cochrane Library databases on August 6, 2022. There were no restrictions on language or publication period.

Study selection was performed by two review authors independently using the following terms: (“Hydroxymethylglutaryl Coenzyme A inhibitor” OR “Hydroxymethylglutaryl Coenzyme A inhibitors” OR “Hydroxymethylglutaryl-Coenzyme A inhibitor” OR “Hydroxymethylglutaryl-Coenzyme A inhibitors” OR “Hydroxymethylglutaryl CoA Reductase Inhibitor” OR “Hydroxymethylglutaryl CoA Reductase Inhibitors” OR “Hydroxymethylglutaryl-CoA Reductase Inhibitor” OR “Hydroxymethylglutaryl-CoA Reductase Inhibitors” OR “HMG CoA Reductase Inhibitor” OR “HMG CoA Reductase Inhibitors” OR “HMG-CoA Reductase Inhibitor” OR “HMG-CoA Reductase Inhibitors” OR Statin OR Statins OR Atorvastatin OR Fluvastatin OR Lovastatin OR Pitavastatin OR Pravastatin OR Rosuvastatin OR Simvastatin) AND (“COVID 19” OR COVID-19 OR “2019 Novel Coronavirus” OR “Coronavirus Disease 2019” OR “Severe Acute Respiratory Syndrome Coronavirus 2” OR “SARS Coronavirus 2” OR “SARS CoV 2” OR “SARS-CoV-2” OR “2019 nCoV” OR “2019-nCoV”) AND (random OR randomized OR randomised OR randomization OR randomly).

Additionally, we used backward snowballing to identify eligible studies from the references of articles identified in the original search. Studies were independently selected by two review authors. Disagreements regarding data collection were resolved by consensus with the participation of a third author. Study screening and selection were performed using the reference manager software Zotero version 6.0.8. We extracted the baseline characteristics and outcome data from the included studies. In addition, we attempted to contact the corresponding author via email to obtain important information that was not reported.

### Quality assessment and statistical analyses

We assessed the potential for bias in all included studies using version two of the Cochrane risk-of-bias tool for randomized trials (RoB 2).^(23)^ Two reviewers independently assessed the risk of bias for each study. Disagreements between the two reviewers were resolved by consensus with a third author. We performed sensitivity analyses for the main outcome(s) by excluding: (i) studies that were judged to be at an overall “high” risk of bias and (ii) studies that were judged to be at an overall “high” risk and “some concerns” of bias.

We evaluated the potential for publication bias using funnel plot visual analyses; however, as the analyzed exposure factor had less than ten studies included in the meta-analysis (k<10), the potential for publication bias was limited. We assessed the strength of the body of evidence using the Grading of Recommendations Assessment, Development and Evaluation (GRADE) system. Two reviewers independently assessed the certainty. Disagreements were resolved by consensus through the participation of a third author. We investigatedthe outcomes of interest “intention-to-treat” by analyzing risk measures and their respective 95% confidence intervals (95%CI). The meta-analysis was independently performed by two authors using the Review Manager (RevMan) software version 5.4. In case of disagreement between the two reviewers, a third author was involved in resolving any discrepancies by consensus.

We computed the pooled odds ratio (OR), with their corresponding 95%CI, for dichotomous outcome data and weighted mean difference (MD) with standard deviation (SD) for continuous outcome data. The results were computed using the DerSimonian and Laird random-effects model. A p value of <0.05 was statistically significant. The heterogeneity of the included studies was assessed using Cochran’s Q test and Higgins and Thompson’s I^2^ index. We regarded a p value of <0.10 as statistically significant when using Cochran’s Q test. We considered I^2^ index values <25% an indication of low heterogeneity.

## RESULTS

### Study selection and characteristics

**Figure 1:**
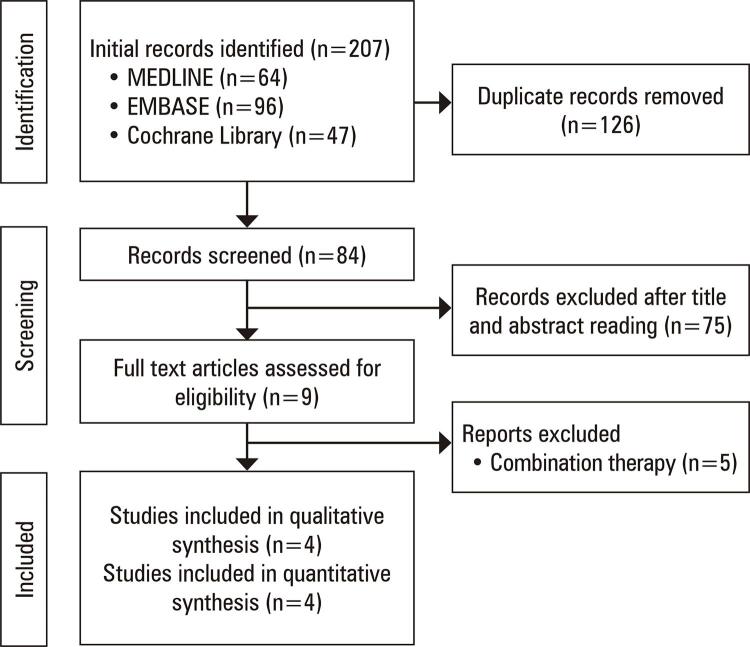
PRISMA 2020 flow diagram of literature search and selection

The initial search yielded 228 studies (Figure 1). After removing duplicates, we screened 84 records, from which we reviewed nine full articles and included four studies. No additional studies that fulfilled the inclusion criteria were found in the screening of the reference lists of the eligible studies.

The pooled data provided a total of 1,231 patients, of whom 610 (49.5%) received statin therapy and 621 (50.5%) received placebo or standard of care. With respect to the criteria used for the diagnosis of COVID-19, three studies^(17,19,20)^ used laboratory-confirmed (virological testing), and one used clinical features only.^(18)^Table 1 summarizes the characteristics of the included studies.

**Table 1:** Baseline characteristics of included studies

Study	Country		Intervention		Control			Follow-up			Study participants, n		Male, n (%)	
Davoodi et al., 2021^(17)^	Iran		Atorvastatin plus lopinavir/ritonavir^†^		Lopinavir/ritonavir			Six days			40		21 (52.5)	
Ghafoori et al., 2022^(18)^	Iran		Atorvastatin plus national protocol medications^‡^		National protocol medications			Until discharge or death			154		78 (50.6)	
INSPIRATION-S, 2022^(19)^	Iran		Atorvastatin^#^		Placebo			30 days			587		331 (56.4)	
RESIST, 2022^(20)^	India		Atorvastatin plus standard of care^≠^		Standard of care			Ten days or until hospital discharge whichever was later			450		327 (74)	

Study	Age^£^, years		Any antiviral therapy, n			Diabetes, n (%)			Hypertension, n (%)			Lung disease, n (%)		
				
	Statin	Control	Statin	Control		Statin	Control		Statin	Control		Statin		Control

Davoodi et al., 2021^(17)^	46.10	45.95	20	20		2 (10)	3 (15)		2 (10)	4 (20)		1 (5)		2 (10)
Ghafoori et al., 2022^(18)^	46^§^	56^f^	76	78		--	--		--	--		--		--
INSPIRATION-S, 2022^(19)^	57	57	233	237		49 (17)	49 (16)		89 (31)	96 (32)		24 (8)		23 (8)
RESIST, 2022^(20)^	51	52	221	219		65 (30)	53 (24)		66 (30)	59 (27)		--		--

^†^ Atorvastatin 40mg plus lopinavir/ritonavir 400/100mg tablets twice daily; ^‡^ atorvastatin 20mg plus lopinavir/ritonavir (400/100mg q12h, orally) with hydroxychloroquine (400mg SD, orally) for the first day followed by lopinavir/ritonavir (400/100mg q12h, orally) for 7–14 days; ^#^ atorvastatin 20mg; ^≠^ atorvastatin 40mg plus hydroxychloroquine (400mg BD x1 day f/b 400mg OD × 4 days in patients at high risk for severe disease; ^£^ mean; ^§^ median.

### Pooled analysis of all studies

**Figure 2:**
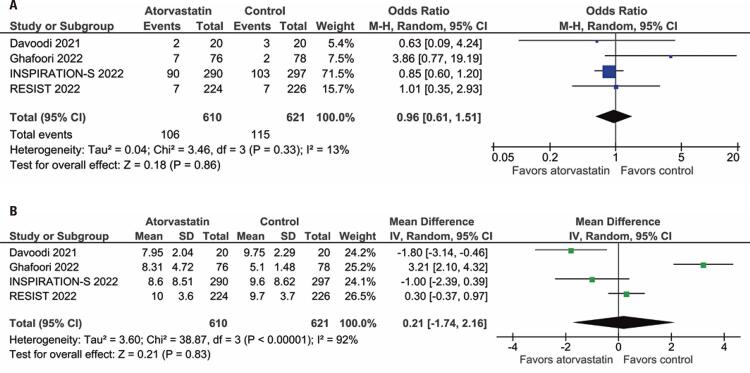
Forest plots depicting. (A) All-cause mortality; (B) Duration of hospitalization among patients hospitalized due to COVID-19 with statin therapy *versus* that with placebo or standard of care

All four studies reported all-cause mortality in the comparison of atorvastatin *versus* placebo or standard of care. There was no significant difference in all-cause mortality between the patients treated with or without statin therapy (OR= 0.96; 95%CI:0.61-1.51; I^2^=13%; p=0.86; Figure 2A). There was also no significant difference between the groups in length of hospital stay (MD= 0.21; 95%CI: -1.74-2.16; I^2^=92%; p=0.8; Figure 2B).

**Figure 3:**
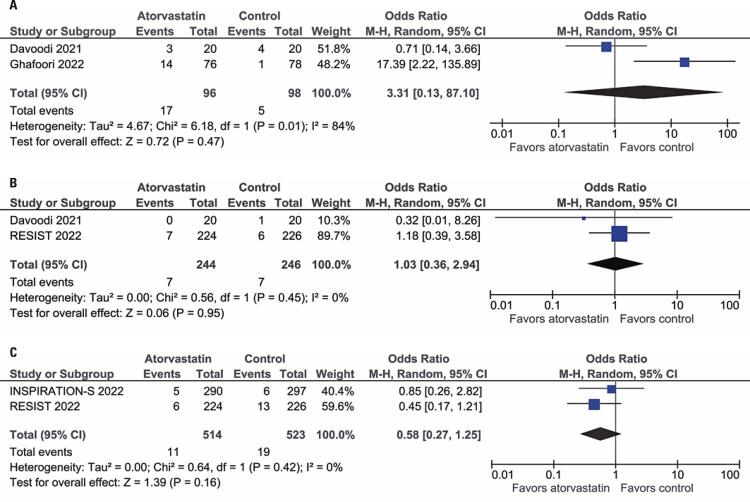
Forest plots depicting (A) Intensive care unit admission; (B) Receipt of invasive mechanical ventilation; (C) Increase in liver enzyme levels among patients hospitalized due to COVID-19 with statin therapy *versus* those in placebo or standard of care

Two studies with a total of 194 patients reported ICU admission for the comparison of atorvastatin *versus* placebo or standard of care. There was no significant difference between the groups with regard to ICU admission (OR= 3.31; 95%CI: 0.13-87.1; I^2^=84%; p=0.47; Figure 3A). In the two studies, with a total of 490 patients, the need for mechanical ventilation was also not significantly different between the groups (OR= 1.03; 95%CI: 0.36-2.94; I^2^=0%; p=0.95; Figure 3B). Finally, the incidence of elevated liver enzyme levels was also similar between the groups (OR= 0.58; 95%CI: 0.27-1.25; I^2^=0%; p=0.16; Figure 3C).

### Sensitivity analyses

The sensitivity analysis excluding studies judged to be at an overall “some concerns” of bias in methodological quality assessment found no difference in all-cause mortality (OR= 1.33; 95%CI: 0.52-3.42; I^2^=23%; p=0.55); length of hospital stay (MD= 0.48; 95%CI: -1.73-2.70; I^2^=95%; p=0.67). Sensitivity analyses excluding studies with “some bias” concerns were not possible for the other outcomes.

### Quality assessment

Davoodi et al.,^(17)^ Ghafoori et al.,^(18)^ and the RESIST trial had an overall “low” risk of bias. We judged INSPIRATION-S at an overall “some concerns” of bias because of “deviations from intended interventions” domain issues (Table 2), since the analysis used to estimate the effect of assignment to intervention was per-protocol.

**Table 2:** Version 2 of the Cochrane risk-of-bias tool for randomized trials of included studies

Study	Bias from randomization process	Bias due to deviations from intended interventions	Bias due to missing outcome data	Bias in measurement of the outcomes	Bias in selection of the reported result	Overall risk of bias
Davoodi et al., 2021	Low	Low	Low	Low	Low	Low
Ghafoori et al., 2022	Low	Low	Low	Low	Low	Low
INSPIRATION-S, 2022	Low	Some concerns	Low	Low	Low	Some concerns
RESIST, 2022	Low	Low	Low	Low	Low	Low

Evidence for all-cause mortality, receipt of invasive mechanical ventilation, and an increase in liver enzyme levels was moderate. We downgraded one level because of imprecision (95%CIs comprised both important clinical benefits and important harm). The certainty of evidence regarding the duration of hospitalization and ICU admission was low. We downgraded another two levels, one due to inconsistency (moderate to considerable unexplained heterogeneity) and one due to imprecision (95%CIs comprised both important clinical benefit and important harm).

## DISCUSSION

In this meta-analysis of four RCTs, we compared statin therapy to no-stating therapy in 1,231 patients hospitalized with COVID-19 and found no significant difference in all-cause mortality, hospital length of stay, ICU admission, need for invasive mechanical ventilation, or increase in liver enzymes between groups.

Although generally considered safe, statin therapy is rarely associated with severe adverse effects in hospitalized patients. Multiple organ failure due to statin rhabdomyolysis has been previously reported, and concurrent administration of drugs that interfere with CYP3A, such as lopinavir and ritonavir, might increase the risk of liver toxicity.^(24)^ Similarly, COVID-19 can cause myalgia and increase liver enzymes, potentially leading to an increased risk of these side effects.^(25)^ The included studies did not provide sufficient data to evaluate safety outcomes.

A meta-analysis of 47 case-control and cohort studies of patients with COVID-19 found no significant effects on ICU admission or all-cause mortality in patients treated with statins, albeit with a reduction in tracheal intubation.^(26)^ However, in hospitalized patients, there was a significant reduction in mortality associated with statin use (OR= 0.54; 95%CI: 0.50-0.58; p<0.001). A prior meta-analysis by Wu et al. also showed a 30% relative reduction in all-cause mortality with statin use versus no statin use in patients with COVID-19.^(27)^

Prior meta-analyses included observational data, which are invariably subject to confounding factors, and might justify the discrepancy between these studies and our results. For example, in one study of 4,252 patients hospitalized with COVID-19, statin non-users had a significantly higher body mass index than that of statin users.^(28)^ Although multivariable analysis models showed similar findings, residual confounding cannot be excluded. The restriction in our study to include only RCTs, along with a large sample size in the pooled data, minimized the risk of confounding.

This study has several limitations. First, a limited number of publications met our inclusion criteria since most data on this topic were observational, and the four RCTs identified included single-site studies of a relatively small number of patients with low event rates. Second, heterogeneity was significant for the outcomes of length of hospital stay and ICU admission. This is not unexpected, given the differences in populations between the studies, burden of comorbidities, criteria for hospital or ICU admissions, and the difference in comparison arms. We aimed to further investigate the origin of this heterogeneity by performing subgroup analyses, which were not possible because of the small number of included papers. However, there was no significant heterogeneity in the main outcome of interest, all-cause mortality. Third, all the included studies evaluated atorvastatin only. The generalizability of our findings to other statins remains unclear. Fourth, in terms of applicability, the four included studies were all conducted from late 2020 to early 2021 and would not have included patients with likely Omicron variant infection. It has become clear that the different variants have significantly differing pathobiologies and risks, including the risk of severe outcomes that might be offset by any therapy, including statins. Therefore, further investigations are necessary.

## CONCLUSION

The results of our systematic review and meta-analysis of randomized controlled trials indicate that in adult hospitalized COVID-19 patients, therapy with statins compared to placebo or standard of care results in no significant difference in all-cause mortality, duration of hospitalization, or admission to the intensive care unit.
